# Computational Prediction of Bacteriophage Host Ranges

**DOI:** 10.3390/microorganisms10010149

**Published:** 2022-01-12

**Authors:** Cyril J. Versoza, Susanne P. Pfeifer

**Affiliations:** 1 Center for Evolution and Medicine, School of Life Sciences, Arizona State University, Tempe, AZ 85281, USA; cversoza@asu.edu; 2 Center for Mechanisms of Evolution, School of Life Sciences, Arizona State University, Tempe, AZ 85281, USA

**Keywords:** bacteriophages, bacterial hosts, bioinformatic tools, host ranges

## Abstract

Increased antibiotic resistance has prompted the development of bacteriophage agents for a multitude of applications in agriculture, biotechnology, and medicine. A key factor in the choice of agents for these applications is the host range of a bacteriophage, i.e., the bacterial genera, species, and strains a bacteriophage is able to infect. Although experimental explorations of host ranges remain the gold standard, such investigations are inherently limited to a small number of viruses and bacteria amendable to cultivation. Here, we review recently developed bioinformatic tools that offer a promising and high-throughput alternative by computationally predicting the putative host ranges of bacteriophages, including those challenging to grow in laboratory environments.

## 1. Introduction

There are approximately 10^31^ viruses on earth [1]—more than stars in the observable universe. The vast majority of this diverse virosphere consists of bacteriophages, i.e., viruses that infect and prey on bacteria. Independently discovered by Frederick William Twort and Félix d’Herelle in the early 1900s [2,3], these abundant biological entities have since been routinely used for a multitude of purposes—ranging from diagnostics [4], to drug design and discovery [5,6], to vaccine development [7], to agriculture [8], to food preservation and safety [9], and to wastewater treatment [10]. 

In order to leverage the bactericidal effects of bacteriophages for these applications, bacterial host ranges (i.e., collections of bacterial species and strains that support the life cycle of the bacteriophage) need to be established. Several experimental techniques allow for the study of bacteriophage–host relationships (such as spot, plaque, and liquid assays, viral tagging, microfluidic PCR, phageFISH, and single-cell genomics [11]). However, they are often time- and labor-intensive, costly, and can be scientifically challenging (e.g., due to inconclusive or absent signs of infection [12]). These approaches are also inherently limited in scope due to both the bacterial cultures used in the experiments—with a limited number of microbial hosts [13,14] and viruses [15,16] being amendable to cultivation—as well as the conditions under which they are performed in the laboratory (such as growth media and temperature [17]). 

Recent advances in sequencing technologies have enabled the discovery and identification of bacteriophages and their hosts from environmental (rather than cultivated) samples, thus providing an important avenue to comprehensively study the natural viral diversity [18,19]. In concert with these technical advances, many bioinformatic approaches have been developed to computationally predict putative bacteriophage host ranges at large scale, based on genomic features shared between bacteriophages and their bacterial hosts through their co-evolution over time. Although predictive by their nature, these tools can highlight the most promising candidates for subsequent experimental work to validate the bacteriophage’s ability to identify and adsorb to the host, as well as to characterize infection cycles, bacteriophage–host interactions, and lysis efficacy. 

In this review, we provide an overview of several available computational host prediction methods, discuss similarities and differences in their design, and provide key considerations when choosing between different approaches.

## 2. Methods to Computationally Predict Bacteriophage Host Ranges

Bioinformatic approaches to computationally predict putative bacteriophage host ranges can be broadly classified into three categories: (i) alignment-based methods based on sequence homology and sequence similarity, (ii) alignment-free methods based on sequence composition and genomic features, and (iii) machine-learning-based methods. 

### 2.1. Alignment-Based Methods

Many factors can impact bacteriophage host specificity. Temperate bacteriophages can integrate their own genomes into that of their bacterial hosts as lysogenic prophages [20]. This process often alters the phenotype of the host, which can lead to an increased fitness (e.g., by providing antibiotic resistance, increasing virulence, producing toxins, or preventing further (super)infections; see review by Touchon and colleagues [21]). At the same time, many bacterial hosts guard themselves against virulent bacteriophages and other invaders by employing a variety of restriction-modification (RM) and clustered regularly interspaced short palindromic repeats (CRISPRs)/Cas (CRISPR-associated protein) strategies [22,23]. In the latter case, a stretch of nucleotides from the invasive genetic material is incorporated into a CRISPR spacer array upon infection (adaptation), and this new spacer is used as a guide to create site-specific cleavages, ultimately leading to the degradation of the invading bacteriophage (immunity) [24]. In both scenarios, the host genome is ultimately altered by, or due to, the invading bacteriophage. 

Alignment-based methods rely on these host–virus shared sequences to computationally predict host ranges from sequence homology (i.e., the common evolutionary ancestry between sequences) and sequence similarity. Many alignment-based methods—including the most prominent example, the Basic Local Alignment Search Tool (BLAST [25])—are straightforward to use, for example by comparing a user-provided viral sequence with those of putative bacterial hosts publicly available in well-maintained (reference) databases. Consequently, the inference of virus–host relationships through alignment-based methods is limited by the comprehensiveness and completeness of the used databases. On the one hand, sequences of bacteriophages that infect a single host not yet present in a database might yield no results; on the other hand, sequences of bacteriophages that exhibit a broad host range might yield multiple results, often ranked by some user-defined criteria (e.g., the overall length of similar sequence) to improve manual/visual dissemination. However, such rankings can also introduce challenges: (i) rankings may change depending on the criteria and thresholds used, (ii) the highest ranked result may not be the most prevalent host (or, in fact, it may not be a host at all), (iii) mosaic bacteriophage genomes may point towards several (equally well supported) related hosts, and (iv) comparable results may arise between distantly related viruses and bacterial species due to spurious alignments or other artifacts. 

To circumvent some of these issues, Zielezinski and colleagues [26] developed a computational tool, Phirbo, that exploits the full range of BLAST results. Phirbo works under the assumption that the similarity between a pair of bacteriophage and host sequences is proportional to the overlap between their independent BLAST searches against the same dataset. Specifically, Phirbo generates two ranked lists from two independent BLAST searches—one using a bacteriophage-reference dataset and one using a host-reference dataset—and compares them using the Ranked-Biased Overlap metric [27], a procedure that has been shown to improve precision compared to several other state-of-the-art host prediction tools [26].

### 2.2. Alignment-Free Methods

Viral and host sequences may lack sequence homology, making them less well-suited for alignment-based methods. In these cases, alignment-free methods offer a promising alternative to infer bacteriophage–host relationships by studying the similarity in patterns of sequence composition, such as codon usage or oligonucleotide (short nucleotide fragment) frequency [11]. Such similarities in patterns of sequence composition are expected from first principles. For example, viruses frequently corrupt the translational machinery of their hosts to synthesize their own viral proteins [28], and this synthesis is generally more efficient if the codon usage patterns of the virus matches that of its host [29,30]. Taking advantage of this relationship, Crane, Versoza and colleagues [31] determined the codon usage bias of 129 mycobacteriophages across 14 putative mycobacterial hosts using COUSIN [32] to obtain important insights into putative mycobacterial host ranges in nature. Bacteriophage genomes can also acquire molecular characteristics of their hosts due to exposure to similar genome-wide mutational pressures, a process referred to as ‘genome amelioration’ [33,34,35]. By matching the nucleotide composition of their hosts, bacteriophages are able to avoid host RM systems that recognize specific tetranucleotides [36]. 

Alignment-free, sequence composition-dependent tools can be categorized by whether the genome-wide signature of a viral sequence is compared to (i) a database of potential hosts (virus–host similarity), or (ii) a database of viruses with known hosts (virus-virus similarity). Examples of the first category include VirHostMatcher [37] which calculates virus–host similarity by comparing oligonucleotide frequencies between the viral sequence and those of potential hosts, and WIsH [38] which calculates virus–host similarity in terms of differences in frequencies of oligonucleotides of a specified length (so-called ‘*k*-mers’). In contrast, HostPhinder [39], an example of the second category, uses virus-virus similarity measures, assuming that similar oligonucleotide usage between viruses indicates shared or closely related hosts.

### 2.3. Machine-Learning Methods

In addition to alignment-based and alignment-free methods, machine-learning (ML) approaches have found a home in bacteriophage research in general [40] and in the prediction of bacteriophage–host interactions specifically [41]. In order to infer virus–host relationships, ML approaches utilize ‘features’, i.e., measurable properties of the object being analyzed such as the nucleotide and amino acid content of the viral genome, amino acid properties, and protein domains (see [42] for a comparison of feature representations). For example, both the Host Taxon Predictor (HTP) [43] and the Prokaryotic virus Host Predictor (PHP) [44] tools use nucleotide features to predict bacteriophage–host interactions, with HTP representing the bacteriophage sequence using absolute and relative frequencies of oligonucleotides as well as nucleic acid types, and PHP using a Gaussian model to predict hosts based on the oligonucleotide frequency differences between viral and host genome sequences. In contrast, PredPHI (Predicting Phage–Host Interactions [45]) identifies putative bacteriophage hosts using a mix of amino acid frequency, chemical composition, and molecular weight as feature representations. Similarly, VirHostMatcher-Net [46] integrates multiple features, including virus-virus similarity, virus–host alignment-free similarity, virus–host alignment-based similarity, and virus–host CRISPR-based similarity, to predict virus–host interactions. BacteriophageHostPrediction [41] uses more than 200 features—ranging from genomic sequences (such as nucleotide and codon frequencies and GC-content), to protein sequences (such as amino acid frequency), to protein secondary structure (such as α-helix and β-sheet frequencies), and to physicochemical properties (such as molecular weight and isoelectric point)–to represent receptor-binding proteins which play a crucial role in determining host specificity by recognizing receptors on the surface of the bacterial host [47]. At a higher level of sequence representation, PHERI [48] infers bacterial hosts from bacteriophage sequences through annotated protein sequence clusters. 

## 3. Bacteriophage–Host Databases

Experimental evidence through bacteriophage isolation and cultivation remains, whenever possible, the gold standard in determining bacteriophage host ranges. However, experimental validation is often time- and labor-intensive. For example, nearly half a decade passed between the initial prediction and concrete experimental evidence that crAssphage–a highly abundant bacteriophage in the human gut microbiome–can infect bacteria of the genus *Bacteroides* [49,50]. As a consequence, information regarding bacteriophage–host relationships remains sparse, with information deposited in the well-established National Center for Biotechnology Information (NCBI) RefSeq and GenBank databases often being either restricted to the genus and/or species level or limited to a handful of samples [51]. The recently developed Viral Host Range database (VHRdb [52])–a web-based tool that integrates host range data as an analysis tool and search engine–aims to collect additional data by allowing researchers to directly share their experimental findings with the scientific community (at the time of writing, 16,715 interactions between 760 viruses and 1923 hosts have been recorded). Given the need of validated training datasets, bacteriophage–host databases such as VHRdb are expected to play a significant role in the development of future ML methods. 

## 4. Method Choice: Key Considerations

### 4.1. Prediction Accuracy

Apart from their underlying algorithms, bacteriophage–host prediction tools also differ in their prediction accuracy, i.e., the percentage of bacteriophages for which the taxonomy of their predicted and known hosts agree [46]. Prediction accuracy can be reported at different taxonomic levels—ranging from the family, genus, and species levels down to the phylum and domain levels. It is thus important to consider which taxonomic levels were measured when selecting the most appropriate tool for any analysis. Methodological differences (such as the type of data included in the benchmarking process) can further contribute to differences in prediction accuracy between tools. Hence, comparisons should ideally be performed using a uniform benchmarking dataset. Using such uniform benchmarking data, Zielezinski and colleagues [26] performed a comparison between a variety of alignment-based, alignment-free, and ML-based host-range prediction tools, demonstrating that tools based on sequence homology generally have a higher predictive accuracy than those reliant on sequence composition similarity (see their Tables 1 and 2).

A challenge faced by researchers working with environmental samples is the non-uniform abundance of microbial species present in a metagenomic sample. As sequencing technologies are optimized for moderate- to high-coverage individual samples, metagenomic samples often result in different read coverage profiles across different genomes [53]. Due to these differences, contigs (a gapless stretch of nucleotide sequence generated by overlapping sequencing reads [54]) obtained from metagenomic samples are frequently short, resulting in genome assemblies that are fragmented and/or incomplete [55]. This is a non-negligible factor in the prediction accuracy of most tools, with short viral contigs (<10 kb) generally experiencing a significant drop in prediction accuracy [26,37,44]. A notable exception in this regard is the tool WIsH, which matches VirHostMatcher’s full-length genome prediction accuracy with merely 3 kb of nucleotide sequence, thus establishing itself as an alignment-free alternative for samples containing short viral contigs.

### 4.2. Usability

Operating system restrictions can be an important aspect in the choice of a suitable bacteriophage–host prediction tool. In order to facilitate both automation and reproducibility, the majority of prediction tools rely on the command line interface (CLI) embedded within UNIX-based operating systems (such as Linux and macOS) (see Table 1). Consequently, users of other operating systems (such as Windows and Chrome OS) will need to either purchase a dedicated machine or install the necessary operating system on an available machine, for example via dual boot or a virtual machine. Windows users can also leverage the Windows Subsystem for Linux (WSL) to allow native Linux programs to run on Windows.

Web-based prediction tools (such as HostPhinder and PHP) offer a valuable alternative. Apart from being user-friendly and intuitive, web-based tools avoid the inconvenience of installation and potential dependency issues, as their only requirement is a compatible browser. However, a major drawback of web-based tools is their cap on input data. For example, while the web-based version of PHP is limited to <100 viruses, the stand-alone version can analyze datasets that are orders of magnitude larger [44]. An additional advantage of many phage–host prediction CLI tools (including Phirbo, WIsH, and VirHostMatcher-Net) is multi-threading which increases the speed of the analyses. 

## 5. Conclusions

Due to their bactericidal effects, bacteriophages are now routinely used for a multitude of biotechnological and clinical purposes, including personalized phage therapy to treat multi-drug resistant infections [56]. Although large-scale bacteriophage banks (such as the Phage Directory [57]) offer a broad range of bacteriophages to the scientific community, the host range that a bacteriophage can infect must be known in order to effectively guide the usage of bacteriophages in these disciplines. Traditional methods to experimentally characterize host ranges–phage isolation and cultivation–remain the gold standard. However, they are time-intensive and thus ill-suited for large-scale analyses. Recently developed computational prediction tools offer a promising alternative, allowing researchers to narrow down the sheer quantity of potential hosts to a limited set that can feasibly (and more cost-efficiently) be tested in a laboratory setting. As tools employ different strategies to predict bacteriophage–host relationships–each with their own advantages and disadvantages, the use of multiple, complementary prediction tools can help to select the most promising candidates, especially for bacteriophages with large host ranges. For example, if time and computational resources permit, a three-way combination of alignment-based, alignment-free, and ML approaches may be used to select those that have been predicted by all three strategies for experimental validation as well as characterization of infection cycles and bacteriophage–host interactions. Although a vast diversity of bacteriophages and bacterial hosts remain to be discovered, advances in genomic databases, machine learning, and high-performance computing have begun to pave the way towards even more sophisticated and accurate computational methods in the near future.

## Figures and Tables

**Table 1:** Computational methods in predicting bacteriophage host ranges.

	Prediction Tool	Input	Output	User Interface	Key Considerations	Reference
**Alignment-based**	Phirbo	two ranked lists (phage and host genomes)	phage–host predictions	CLI (Python)	Linux and macOS multi-threading support	[26]
**Alignment-free**	HostPhinder	phage FASTA file	predicted hosts	web-based	not limited to any OS	[39]
	VirHostMatcher	phage FASTA file host FASTA file taxonomy text file	dissimilarity index phage–host pairs	CLI (Python)	Linux, macOS, Windows	[37]
	WIsH	phage FASTA file host FASTA file	predicted hosts	CLI (C++)	Linux and macOS multi-threading support	[38]
	BacteriophageHostPrediction	phage FASTA file	predicted hosts	CLI (Python)	Linux and macOS	[41]
**Machine-learning-based**	Host Taxon Predictor (HTP)	phage FASTA file	Predicted host lineages	CLI (Python)	Linux and macOS	[43]
	
	Prokaryotic virus Host Predictor (PHP)	phage FASTA file	predicted hosts	CLI (Python); web-based	Linux and macOS user-defined training models	[44]
	PredPHI	protein sequences (phage–host pairs)	phage-host predictions	CLI (Python)	Linux and macOS	[45]
	PHERI	phage FASTA file	predicted hosts predicted shared genes protein sequence clusters	CLI (Python)	Linux and macOS	[48]
	VirHostMatcher-Net	phage FASTA file	predicted hosts	CLI (Python)	Linux and macOS multi-threading support	[46]

CLI, command line interface; OS, operating system; FASTA file, text file representing nucleotide or amino acid sequences.

## Data Availability

Not applicable.
